# Research on Anal Squamous Cell Carcinoma

**DOI:** 10.3390/cancers14010042

**Published:** 2021-12-23

**Authors:** Krishan R. Jethwa, Christopher L. Hallemeier

**Affiliations:** Department of Radiation Oncology, Mayo Clinic, Rochester, MN 55905, USA

Anal canal and peri-anal squamous cell carcinomas (ASCCs) are relatively rare cancers that affect approximately 8000 patients per year in the United States. The rate appears to be rising, which is potentially attributable to the increasing incidence of oncogenic Human Papilloma Virus (HPV) infection. Historically, patients were treated with abdominoperineal resection with resulting permanent colostomy dependence. Through landmark efforts and sequentially designed clinical trials, the majority of patients with localized disease are now effectively treated with radiotherapy and chemotherapy, or in select cases, sphincter-preserving surgery, with favorable outcomes. However, continued efforts are needed to gain a better understanding of ASCC disease biology and to continue improving cancer control while maximizing the quality of life for patients. The purpose of this Special Issue is to discuss the historical perspectives of surgery, radiotherapy, and systemic therapy; the evolution of therapy over time; and current areas of investigation to advance our understanding of the multi-disciplinary management of patients with ASCC.

This Special Issue consists of six articles that provide an in-depth overview of contemporary practice and future areas of investigation. First, Dee et al. [1] and Possiel et al. [2] discuss the evolution of the role of radiotherapy for ASCC, providing key insights into advanced radiotherapy technologies such as the use of intensity-modulated radiotherapy and proton therapy. Carr et al. [3] and Wind et al. [4] discuss the evolution of systemic management for ASCC, including contemporary practice for both locoregional disease and metastatic disease, along with the potential inclusion of induction chemotherapy for select patients with locoregionally advanced disease. Furthermore, they provide valuable suggestions into the future of ASCC management, including the incorporation of immune checkpoint inhibition and other targeted therapies. Lefèvre et al. [5] discuss the novel quantification and dynamics of HPV circulating tumor DNA as a prognostic indicator for patients receiving chemoradiotherapy for ASCC. Finally, Spehner et al. [6] provide a broad summative overview highlighting novel areas of future investigation to help improve outcomes for patients.

In conclusion, this Special Issue presents an in-depth discussion of the evolution of therapy for patients with ASCC, highlights the importance of multidisciplinary collaboration, and introduces numerous areas of active investigation to help improve outcomes for our patients.
